# HPLC-Fluorescence Detection Method for Concurrent Estimation of Domperidone and Naproxen. Validation and Eco-Friendliness Appraisal Studies

**DOI:** 10.1007/s10895-022-03067-1

**Published:** 2022-12-20

**Authors:** Karin M. Guirguis, May M. Zeid, Rasha A. Shaalan, Tarek S. Belal

**Affiliations:** 1 Pharmaceutical Chemistry Department, Faculty of Pharmacy, Pharos University in Alexandria, Canal El-Mahmoudia, Alexandria, Egypt; 2 Pharmaceutical Analytical Chemistry Department, Faculty of Pharmacy, University of Alexandria, Elmessalah, Alexandria, 21521 Egypt

**Keywords:** Domperidone, Naproxen, HPLC, Fluorimetric detection, Greenness assessment

## Abstract

This work demonstrates a simple and reliable HPLC method with fluorimetric detection for simultaneous estimation of domperidone (DOM) and naproxen (NAP). Successful chromatographic separation was accomplished using Inertsil ODS C18 column (5 μm, 4.6 × 150 mm) with gradient elution of the mobile phase consisting of 0.01 M phosphate buffer (pH 5.5) solution and acetonitrile. The gradient elution started with 25% acetonitrile increased linearly to 65% in 5 min, then kept at this percentage till the end of the run. The mobile phase was pumped at a flow rate of 1.0 mL/min. The excitation wavelength at 284 nm was found suitable for both DOM and NAP since it corresponds to a maximum for the minor component DOM and measurable excitation for NAP, while using 316 and 355 nm as emission wavelengths for DOM and NAP, respectively. Peaks eluted with excellent resolution at retention times 4.4 and 6.3 min for DOM and NAP, respectively. Performance of the proposed method was tested according to ICH guidelines in regard to linearity, ranges, precision, accuracy, robustness, detection and quantitation limits. Calibration curves were linear in the ranges of 0.8–3.6 and 1.0–2.5 µg/mL for DOM and NAP respectively with correlation coefficients not less than 0.9996. The validated method was successfully applied to the analysis of DOM and NAP in their laboratory prepared tablets resembling the commercial dosage form, and assay results were favorably compared with a published reference HPLC method. The method’s greenness was assessed using the Analytical Eco-Scale and the novel Analytical Greenness metric (AGREE).

**Supplementary Information:**

The online version contains supplementary material available at 10.1007/s10895-022-03067-1.

## Introduction

**Fig. 1:**
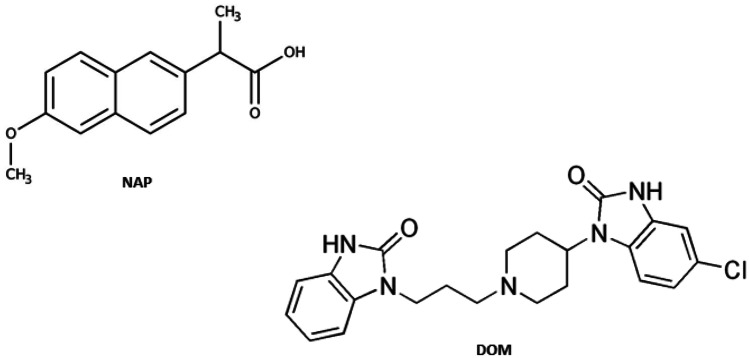
Chemical structures of naproxen (NAP) and domperidone (DOM)

Naproxen (NAP), a propionic acid derivative, is a non-steroidal anti-inflammatory drug (NSAID) [1]. It is chemically (2S)-2-(6-methoxynaphthalen-2-yl) propanoic acid (Fig. 1) [2]. Ankylosing spondylitis, osteoarthritis, and rheumatoid arthritis are among the musculoskeletal and joint conditions for which NAP is prescribed. It is also used to treat dysmenorrhea, migraines, post-operative discomfort, soft-tissue disorders, acute gout and fever [1]. It works by reversibly inhibiting both the COX-1 and COX-2 enzymes. As a result, prostaglandin synthesis is inhibited [3].

Domperidone (DOM) is chemically 5-chloro-1-[1,3- (2,3-dihydro-2-oxo-1H-benzimidazol-1yl) propyl)-4-piperdinyl-1,3-dihydro-2Hbenzimidazol- 2-one] (Fig. 1). It is a dopamine antagonist with similar effects and usage to metoclopramide. It is used as an antiemetic to alleviate nausea and vomiting in the short term [1]. Domperidone does not cross the blood–brain barrier, hence it has less central nervous system side effects than other dopamine antagonists.

A one-of-a-kind formulation combining NAP 250 mg and DOM 10 mg is accessible within the worldwide market. Such a composition may be an issue for investigation and requires the usage of a sensitive approach, particularly for evaluating DOM at such low concentration. Several publications in the literature point to UV spectrophotometry as an important tool for developing analytical methods for the simultaneous estimation of NAP and DOM. Described spectrophotometric methods include Q-analysis [4, 5], simultaneous equations method [2, 4], absorbance ratio method [6], first order derivative method [5], isoabsorptive point, ratio subtraction, ratio difference, and mean centering methods [7]. According to a review of the literature, RP-HPLC methods with UV detection [8–11] and PDA detection [3] for the simultaneous quantification of NAP and DOM have been reported. Moreover, the studied literature described an HPTLC method for the simultaneous quantitation of domperidone maleate and naproxen sodium in bulk drug and formulation [12].

NAP and DOM are characterized by their native fluorescence. Accordingly, several fluorescence-based methods have been applied for the analysis of both compounds separately. It can be noticed that numerous studies have been published on the determination of NAP based on its fluorescence properties. These include a magnetic solid phase extraction method followed by liquid chromatography with a fluorescence detector for its analysis in human urine [13], submicellar liquid chromatography with fluorescence detection in plasma and brain samples [14], and spectrofluorometric determination of NAP in tablets [15]. NAP was also analyzed simultaneously with diphenhydramine using first derivative synchronous spectrofluorimetry [16], with diflunisal using synchronous luminescence spectrometry [17], with salicylate in human serum using direct spectrofluorometry assisted by chemometric analysis [18] and with salicylic acid in human serum using second-derivative synchronous fluorescence spectroscopy [19]. Similarly, DOM was determined in human plasma using high performance liquid chromatography with fluorescence detection [20]. The simultaneous determination of DOM in the binary mixture with itopride was achieved by fourth-derivative synchronous spectrofluorimetry and HPLC with fluorescence detection [21]. and by second-derivative synchronous fluorometric method for its analysis with cinnarizine [22]. Additionally, an HPLC assay with fluorescence detection was employed for the determination of DOM and three major metabolites [23].

No reported methods were found in the literature for the simultaneous determination of both drugs using any fluorescence-based method. Hence, a facile, selective, and reliable HPLC-fluorescence detection (HPLC-FD) technique for the simultaneous analysis of NAP and DOM in bulk form and in their synthetic mixture was implemented. The suggested method was systematically validated and tested for eco-friendliness using some recently introduced greenness assessment tools.

## Experimental

### Instrumentation

The HPLC-FD system consisted of Agilent 1200 series (Agilent Technologies, Santa Clara, CA, USA) (auto-injector, quaternary pump, vacuum degasser and fluorescence detector G1321 B) connected to a computer loaded with Agilent ChemStation Software. The column used was Inertsil ODS C18 column (5 μm, 4.6 × 150 mm).

### Materials and Chemicals

DOM and NAP were kindly supplied by Pharco Pharmaceuticals Company, Alexandria, Egypt. HPLC-grade acetonitrile (Fisher Scientific, UK), HPLC-grade methanol (Fisher Scientific, UK), analytical grade of disodium hydrogen orthophosphate dihydrate (SD Fine Chem limited, Mumbai, India), ortho-phosphoric acid (SD Fine Chem limited, Mumbai, India) and high purity distilled water were used. The pharmaceutical preparation assayed in the study was Laboratory-made tablets for domperidone and naproxen mixture using tablet fillers (maize starch, microcrystalline cellulose "Avicel", magnesium stearate and colloidal silica "Aerosil") supplied from Pharco Pharmaceuticals Company, Alexandria, Egypt.

### Preparation of Standard and Sample Solutions

#### Preparation of Standard Stock Solutions

NAP (1000 μg/mL) and DOM (1000 μg/mL) stock solutions were separately prepared by dissolving accurate weights of 25 mg drug in HPLC-grade methanol in two 25 mL volumetric flasks, then diluting to volume with the same solvent. Sonication for few minutes was applied to aid the solvation.

#### Preparation of Sample Stock Solution

The formulation assayed in the study was laboratory-made tablets each containing 250 mg NAP and 10 mg DOM. Inactive ingredients used in the preparation of tablets were maize starch, microcrystalline cellulose "Avicel", magnesium stearate and colloidal silica "Aerosil". Ten tablets were accurately massed, crushed and homogenized. An amount of the tablets powder equivalent to 250 mg NAP and 10 mg DOM was weighed. The active ingredients were extracted by 20 mL of methanol, sonicated for 30 min then filtered into a 100 mL volumetric flask. Two portions of 25 mL methanol were used to wash the residue and then added to the filtrate. Finally, the solution was diluted with methanol to reach a final concentration of 2500 µg/mL NAP and 100 μg/ml DOM. Further dilution of the stock was needed to obtain a measurable amount of NAP; therefore 1 mL was taken from the sample stock solution and transferred to a 100 mL volumetric flask and dilution was done using methanol to reach a final solution of 25 µg/mL NAP.

### General Procedure

#### Chromatographic Conditions

The analytical column used was Inertsil C18 (5 μm, 4.6 × 150 mm). The mobile phase consists of 0.01 M phosphate buffer pH 5.5 and acetonitrile HPLC-grade. The phosphate buffer was prepared by weighing 0.8898 g disodium hydrogen orthophosphate dihydrate in 500 mL distilled water and adjusted to pH 5.5 with diluted ortho-phosphoric acid. The separation was achieved in 8 min with the linear gradient program illustrated in Table 1. The flow rate was 1.0 mL/min throughout the run. The injection volume was 20 μL. The eluant was monitored by the fluorescence detector, and chromatograms were extracted at λ_em_ 316 nm and 355 nm for DOM and NAP, respectively after excitation at 284 nm. All determinations were performed at 25 ºC.

**Table 1:** Gradient program used in the study

**Time (min)**	**0.01 M phosphate buffer (pH 5.5) %**	**Acetonitrile %**	**Flow (mL/min)**
0	75	25	1.0
5	35	65	1.0
8	35	65	1.0

#### Construction of Calibration Graphs

The working solutions were prepared by dilution of aliquots of NAP stock solution (10.0 – 25.0 µL) and DOM stock solution (8.0 -36.0 µL) in 10-mL volumetric flasks with the mobile phase to reach the concentration ranges 1.00 – 2.50 and 0.80 – 3.60 μg/mL for NAP and DOM, respectively. Triplicate injections were made for each concentration and chromatographed under the previously described HPLC conditions. Peak areas were recorded at λ_em_ 355 nm for NAP and λ_em_ 316 nm for DOM. The peak areas were plotted against the corresponding concentrations to construct the calibration graphs.

#### Procedure for Pharmaceutical Preparation

Aliquots of the appropriate stock sample solution were diluted with mobile phase to reach final concentrations within the prescribed ranges, then chromatographed using the previously stated chromatographic conditions, and recovered concentrations were computed using the calibration graphs.

## Results and Discussion

### Optimization of Chromatographic Conditions

An effective routine quality control analysis of a binary combination of NAP and DOM was developed using a gradient liquid chromatographic technology with fluorimetric detection. The proposed method was deliberately designed to separate the selected drugs with adequate resolution, sufficient peak symmetry, and a reasonable analysis time. Various trials were conducted to optimize both the stationary and mobile phases aiming to accomplish this target. A couple of reversed phase columns were tested for optimization of the stationary phase. These include Eclipse XBD C18 (5 μm, 4.6 × 150 mm) and Inertsil ODS C18 (5 μm, 4.6 × 150 mm). Both columns managed to resolve the two analytes. Because of the bad peak shape obtained when using Eclipse XBD C18 column, the Inertsil ODS C18 (5 μm, 4.6 × 150 mm) became the column of choice for this study.

Several mobile phases were assessed using various proportions of different aqueous phases. The principal mobile phase combination was acetonitrile and 0.01 M phosphate buffer (pH 5.5) solution. As an organic phase, acetonitrile was the best choice because when replaced by methanol the analyte peaks appeared to be very broad. Phosphate buffer solution was substituted by water where the peaks were overlapped, broad and tailed. Another experiment with orthophosphoric acid (pH 3) in place of phosphate buffer revealed that the peaks were tailed. The impact of phosphate buffer pH was studied in the range 2.5–6.5. The peaks at pH 2.5 were very broad and tailed. Furthermore, phosphate buffer pH 3 and 3.5 yielded the same results as pH 2.5 in addition to extending the run time. pH 4, 4.5, 5 and 6 were also tested and they did not show satisfactory results regarding the peak shape as peak broadening and tailing remained a problem. Consequently, it was found that 0.01 M phosphate buffer (pH 5.5) showed excellent peak shape and reasonable run time. Gradient elution was used to separate the two peaks in appropriate retention time, starting with high aqueous and low acetonitrile percentages, and increasing the organic modifier linearly up to a specified value. Several gradient programs were tried. A gradient system was used to achieve the optimal compromise between appropriate resolution, suitable retention times, and tolerable peak asymmetry, starting with 25% (by volume) acetonitrile and ramping up linearly to 65% in 5 min, then remaining at this percentage until the end of the run (Table 1). Other gradient elutions were tried, but they resulted in peak broadening and tailing along with the increase in run time. Throughout the run, the flow rate was maintained at 1.0 mL/min, and the column temperature was set to 25 °C.

**Fig. 2:**
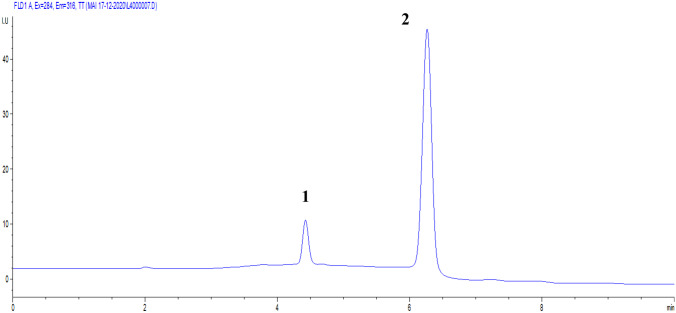
HPLC chromatogram at λ_ex_ 284 nm and λ_em_ 316 nm of 20 μL injection volume of a mixture containing: (**1)** 2.0 μg/mL DOM and (**2**) 2.0 μg/mL NAP

Fluorescence detection was used to detect the analytes in this mixture. NAP exhibits excitation wavelength at 271 nm and emission wavelength at 355 nm. On the other hand, DOM exhibits excitation wavelength at 284 nm and emission wavelength at 316 nm. The major component of the mixture (NAP) showed higher fluorescence in comparison to the minor component (DOM). Consequently, the excitation wavelength at 284 nm was found suitable for the separation and quantitation of both NAP and DOM since it corresponds to a maximum for the minor component (DOM) and measurable absorbance for NAP with using both drugs emission wavelengths for quantitation. Changing the emission wavelength in a time-programming was tried to allow the quantitation of both analytes in the same chromatogram; however, it affected the chromatogram’s baseline, therefore quantitation was obtained in two separate chromatograms using both emission wavelengths. Since NAP (major component) fluorescence was much higher than DOM (minor component), increasing the sensitivity of the detector was tested by raising the detector’s gain till 14 in order to get a compromise in favor of the minor component (DOM). However, this showed a massive distortion in the baseline so the detector was used with its normal sensitivity (Gain 10). The analytes were separated satisfactorily under the previously indicated chromatographic settings. A sample chromatogram for separating this complex mixture is shown in Fig. 2.

The elution order of the studied mixture is DOM followed by NAP at retention times 4.4 and 6.3 min respectively. Retention times, capacity factors, theoretical plates, resolution and other system suitability criteria have all been computed and found to be satisfactory. It's worth noting that the resolution value between the two drugs was 8.5, indicating that the baseline separation was excellent (Table 2).

**Table 2:** System suitability parameters for the separated compounds in this study

**Compound**	**t**_**R**_ **± SD (min)**	**Capacity factor** **(k′)**	**Theoretical plates** **(N)**	**Tailing factor**	**Selectivity (α)**	**Resolution (Rs)**
**DOM**	4.43 ± 0.002	1.22	10,621	1.11		
**NAP**	6.27 ± 0.004	2.14	9243	0.96	1.42	8.50

### Validation of the Proposed Method

The proposed HPLC method was validated using the International Conference on Harmonization (ICH) guidelines for analytical process validation [24].

#### Linearity and Concentration Ranges

The suggested HPLC procedure's linearity was tested using a series of varied concentrations for each of the two analytes. The calibration data were treated with least squares to create the linear regression equations. Table 3 shows the performance data as well as statistical merits such as linear regression equations, concentration ranges, correlation coefficients, standard deviations of the intercept (S_a_), slope (S_b_), and standard deviation of residuals (S_y/x_). As can be seen from the correlation coefficient values (> 0.9995), regression analysis revealed good linearity. Additionally, deviation around the slope can be also assessed by calculation of the RSD% of the slope (S_b_%) which were found to be less than 1.57%. Figures S1 and S3 (in supplementary file) elucidate the calibration plots of peak area data versus the corresponding concentrations of DOM and NAP, respectively.

**Table 3:** Analytical parameters for the determination of NAP–DOM mixture using the proposed HPLC-FD method

**Parameter**	**NAP**	**DOM**
Concentration range (μg/mL)	1.00 – 2.50	0.80 – 3.60
Intercept (a)	-1.506	1.749
S_a_^a^	5.301	0.741
Slope (b)	195.418	25.887
S_b_^b^	3.066	0.311
RSD% of the slope (S_b_%)	1.569	1.201
Correlation coefficient (r)	0.99963	0.99957
S_y/x_^c^	3.746	0.806
F^d^	4.06 × 10^3^	6.94 × 10^3^
Significance F	8.51 × 10^–6^	2.02 × 10^–10^
LOD^e^ (μg/mL)	0.086	0.179
LOQ^f^ (μg/mL)	0.286	0.598

^a^ Standard deviation of the intercept

^b^ Standard deviation of the slope

^c^ Standard deviation of residuals

^d^ Variance ratio, equals the mean of squares due to regression divided by the mean of squares about regression (due to residuals)

^e^ Limit of detection

^f^ Limit of quantification

Furthermore, the fitting of data to the regression line was evaluated by the residuals plots of the regression data of the two drugs. Figures S2 and S4 (in supplementary file) show the vertical deviations of the experimental data points from the least-square line (Y_observed_ – Y_calculated_) for DOM and NAP, respectively. In a random manner, some points deviate above and some deviate below the line. The analysis of variance (ANOVA) test can also be used to support linearity. The most significant statistical parameter in this test is the F-value which is the ratio of the mean of squares due to regression divided by the mean of squares due to residuals. High F values show an increase in the mean of squares due to regression and a decrease in the mean of squares due to residuals. The greater the mean of squares due to regression, the steeper is the regression line. The smaller the mean of squares due to residuals, the less is the scatter of experimental points around the regression line. As a result, regression lines with high F values (low significance F) perform much better than those with lower F values. Both the r and F statistical parameters have high values in good regression lines.

#### Detection and Quantification Limits

The concentration of analyte with a signal-to-noise ratio of 3:1 is defined as the limit of detection (LOD). The ratio taken into account for the limit of quantification (LOQ) is 10:1. The signal-to-noise ratio approach was used to obtain the LOD and LOQ values of NAP and DOM, which are presented in Table 3. The suggested HPLC method's sensitivity is assured by both LOD and LOQ values.

#### Accuracy and Precision

Three replicate determinations for each concentration within one day were used to assess the suggested method's within-day (intra-day) precision and accuracy at three concentration levels for each analyte. Similarly, the precision and accuracy of between-day (inter-day) results were investigated by evaluating the same three concentrations for each drug in three replicates across three days. The corresponding regression equations were used to calculate recoveries, which were found to be satisfactory. The percentage relative standard deviation (RSD %) and percentage relative error (E_r_ %) were both less than 2.0 percent, indicating that the suggested method for determining analytes in bulk form is highly reproducible and accurate (Table 4).

**Table 4:** Precision and accuracy for the analysis of NAP and DOM in bulk form using the proposed HPLC-FD method

		**Nominal value (μg/mL)**	**Found ± SD**^**a**^ **(μg/mL)**	**RSD(%)**^**b**^	**Er(%)**^**c**^
**NAP**	**Within-day**	1.00	1.00 ± 0.003	0.30	0.00
		2.00	2.03 ± 0.004	0.20	1.50
		2.50	2.48 ± 0.019	0.77	- 0.80
	**Between-day**	1.00	1.02 ± 0.018	1.76	2.00
		2.00	2.02 ± 0.007	0.35	1.00
		2.50	2.47 ± 0.000	0.00	- 1.20
**DOM**	**Within-day**	1.20	1.20 ± 0.007	0.58	0.00
		2.80	2.85 ± 0.003	0.11	1.79
		3.20	3.16 ± 0.028	0.89	- 1.25
	**Between-day**	1.20	1.21 ± 0.006	0.50	0.83
		2.80	2.85 ± 0.003	0.11	1.79
		3.20	3.23 ± 0.065	2.01	0.94

^a^ Mean ± standard deviation for three determinations

^b^ % Relative standard deviation

^c^ % Relative error

#### Robustness

Robustness was evaluated by making small changes in acetonitrile content in the mobile phase (± 2%), flow rate (± 0.05 mL/min), column temperature (± 5º C), pH of the buffer (± 0.5 units) and working wavelengths (± 1 nm) and recording the chromatograms of a standard mixture of both target analytes. The retention times of the eluting peaks, as well as the measured responses (peak areas) of NAP and DOM, were not affected by these changes. Table 5 shows the effects of the investigated variations on the analytes' retention times and peak areas. Furthermore, the resolution between them was unaffected by these minor experimental changes. For the baseline separation of closely eluted peaks, a resolution of 1.5 is usually regarded acceptable [25]. The resolution between the two peaks was always more than or equal to 8.03, implying excellent baseline separation.

**Table 5:** Evaluation of robustness of the proposed HPLC-FD method

**Parameter**	**Compound**	**Retention time ± SD**	**Resolution** **(Rs ± SD)**	**Peak area ± SD** **DOM at**_**λem**_ **316 nm and NAP at**_**λem**_ **355 nm**	**RSD%**
**Acetonitrile percentage in the mobile phase (± 2%)**	**DOM**	4.38 ± 0.255		49.87 ± 1.504	3.02
	**NAP**	6.36 ± 0.250	8.07 ± 0.252	331.67 ± 1.528	0.46
**Flow rate** ** ± 0.05 mL/min**	**DOM**	4.37 ± 0.154		50.77 ± 1.443	2.84
	**NAP**	6.37 ± 0.253	8.28 ± 0.069	332.67 ± 0.351	0.11
**Column temperature** ** ± 5 °C**	**DOM**	4.42 ± 0.008		50.30 ± 1.212	2.41
	**NAP**	6.41 ± 0.093	8.26 ± 0.053	331.73 ± 1.206	0.36
**Working wavelength (λex 284 nm) ± 1 nm**					
	**DOM**	4.34 ± 0.077		49.70 ± 1.706	3.43
	**NAP**	6.32 ± 0.041	8.03 ± 0.208	332.87 ± 0.808	0.24
**pH ± 0.5 units**	**DOM**	4.47 ± 0.207		55.53 ± 1.343	2.42
	**NAP**	6.14 ± 0.931	11.27 ± 4.969	316.90 ± 0.854	0.27

#### Specificity

The peak purity of NAP and DOM was evaluated by comparing their respective spectra (Figs. S5 and S6 in supplementary file). The method’s specificity was proven by the acceptable peak purity and correlation values (r value more than 0.999) indicating no interference in the quantitation of NAP and DOM. Moreover, the diluent (mobile phase) and placebo solutions show no interference in the quantitation of NAP and DOM as proved by their chromatograms shown as Figs. S7 and S8 in supplementary file.

#### Stability of Solutions

The stability of NAP and DOM working solutions as well as the sample solutions in mobile phase was examined, and no chromatographic changes were observed within 6 h at room temperature (22 ± 2 °C), subsequent peak areas obtained during a time period of 6 h show no significant change giving evidence of solution stability, this applies for working solutions as well as for sample solutions, data are tabulated in Table S1 in supplementary file. Furthermore, when refrigerated at 4 °C, the stock solutions remained stable for at least 4 weeks. During these times, the drug retention times and peak areas remained unchanged, and no significant degradation was observed.

### Assay of Laboratory-Prepared Tablets Dosage Form

**Fig. 3:**
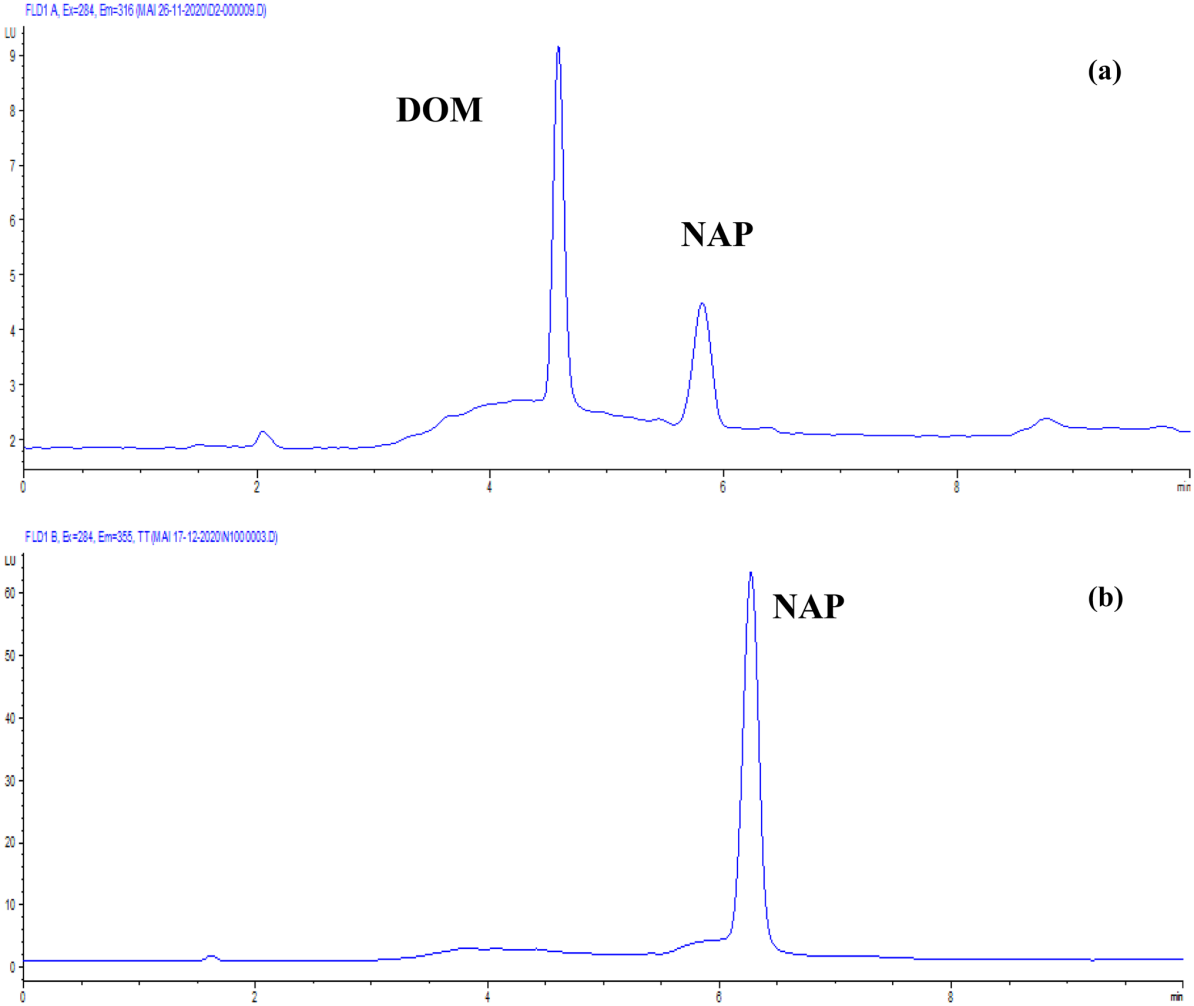
HPLC chromatograms at λex 284 nm of solution containing (**a**) 25 µg/mL NAP and 1 µg/mL DOM at λem 316 nm and (**b**) 2 µg/mL NAP and 0.08 µg/mL DOM at λem 355 nm obtained from laboratory prepared tablet solution

The proposed HPLC-FD method was used to analyze this drug combination as a synthetic mixture due to the lack of its commercial pharmaceutical formulation in the Egyptian market. Figure 3 shows representative chromatograms obtained from the sample solution.

The active ingredients eluted at their specific retention times. Recoveries were calculated using external standard solutions. As seen by the % recovery, SD, and RSD % values, the analysis revealed appropriate accuracy and precision (Table 6). Furthermore, for the simultaneous evaluation of NAP and DOM in their combined dosage form, a previously published HPLC method [10] was used as reference method. The proposed HPLC-FD method's recovery data were statistically compared to the reported method's using the Student's t- and variance ratio F-tests. At the 95 percent confidence level, the calculated values did not surpass the theoretical ones in both tests, indicating that there were no significant differences between the recoveries obtained using the suggested method and those obtained using the reported method (Table 6). The new approach depending on the fluorescence property of NAP and DOM is clearly suitable to the analysis of this binary mixture with a concentration ratio NAP:DOM (25:1) equivalent to the marketed tablet dosage form with a reasonable level of selectivity, accuracy, and precision, as shown by these results.

**Table 6:** Application of the proposed HPLC-FD method to the analysis of NAP-DOM synthetic mixture

**Parameters**	**Proposed method**		**Reported HPLC method [**10**]**	
	**NAP**	**DOM**	**NAP**	**DOM**
**%Recovery ± SD**^**a**^	100.52 ± 0.247	99.96 ± 0.661	99.93 ± 0.351	100.23 ± 0.208
**RSD%**^**b**^	0.247	0.661	0.351	0.208
**t**	2.355	0.672		
**F**	2.014	10.080		

^a^ Mean ± standard deviation for five determinations

^b^ % Relative standard deviation

Theoretical values for t and F at *P* = 0.05 are 2.77 and 19.00, respectively

### Greenness Assessment and Comparison of the Developed Method and Reported Methods

Before developing analytical procedures, it is important to evaluate their impact on the environment. To get a more meaningful comparison and logical ranking of various analytical approaches in terms of their eco-friendliness characteristics, the adoption of various greenness evaluation tools is encouraged. In our study, two methods were used to evaluate the greenness level, namely the Analytical Eco-Scale [26] and the novel Analytical Greenness metric (AGREE) [27]. Such tools have been expensively applied to measure level of greenness in several recent pharmaceutical analysis reports [28–30].

The analytical Eco-scale has the benefit of being both semi-quantitative and easily calculated. The Eco-Scale score is calculated by deducting penalty points from a base score of 100 for each component (reagent amount and nature, occupational hazard, energy consumption, and amount of waste) that does not meet the criteria for an ideal green analysis. Excellent green analysis is specified by scores greater than 75, good green analysis is defined by scores between 50 and 75, and insufficient green analysis is specified by scores lower than 50. With a determined analytical Eco-Scale score of 92 (Table 7), the proposed method might be regarded as having excellent green performance with little to no detrimental effects on the environment or human health.

**Table 7:** Penalty points of the proposed method and some reported methods according to the Analytical Eco-scale

**Parameter**	**Proposed HPLC -FD**	**Reported HPLC–PDA [**3**]**	**Reported HPTLC [**12**]**	**Reported UV Spectrophotometry [**4**]**
Acetonitrile	4	4	-	-
Hydrogen orthophosphate dihydrate	0	-	-	-
Toluene	-	-	6	-
Methanol	-	-	6	6
Acetone	-	-	4	-
Energy	1	1	1	0
Occupational hazards	0	0	0	0
Waste	3	3	3	3
**PPs**	**8**	**8**	**20**	**9**
**Eco-scale score**	**92**	**92**	**80**	**91**

The new, inclusive, and enlightening AGREE tool was used to assess the methodologies' greenness further. This tool has the benefit of taking into account the green analytical chemistry (GAC) 12 guiding principles. As a result, a thorough assessment of each method's greenness was achieved, and a systematic distinction between the newly developed HPLC-FD method and previously reported chromatographic and spectrophotometric methods regarding their green characteristics was produced (Table 8). The AGREE tool demonstrated that the proposed method showed almost equivalent AGREE analytical score of 0.81 when compared with some reported methods. Both the reported HPLC–PDA method [3] and the spectrophotometric method [4] received an AGREE score of 0.82. The reported HPTLC method [12] acquired the least AGREE analytical score of 0.77; this is due to the use of organic solvents, namely toluene, methanol and acetone as mobile phase, while the proposed method’s mobile phase consisted of a mixture of aqueous phosphate buffer and acetonitrile. As a result of combining the two tools, we discovered that our HPLC-FD method is a green method with minimal risk to human health or the environment.

**Table 8:**
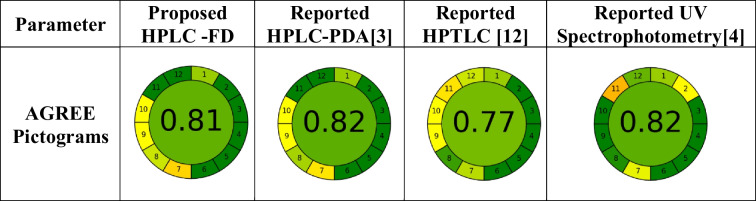
Greenness assessment and comparison of the proposed method and some reported methods using AGREE matrix

## Conclusion

A novel HPLC-FD method was developed for the simultaneous estimation of NAP and DOM in bulk and laboratory-prepared tablets in which the active agents were present in concentrations resembling the marketed dosage form. The proposed assay method was shown to be a green, sensitive, simple, quick, and cost-effective. With a ratio of (NAP: DOM = 25:1) in the dosage form, the proposed method proved to be advantageous for the sensitive determination of DOM in its low concentration compared to NAP. The method was validated as per ICH guidelines. All metrics including linearity, precision, accuracy, and robustness met the ICH recommendations. Additionally, two different metrics (Analytical Eco-Scale and AGREE) were used to evaluate the greenness of the proposed method and compare it to some other reported methods. The suggested HPLC-FD method confirmed to be of equal greenness to some of the published methods. As a result, the suggested method can be easily used for routine NAP and DOM analysis in combined dosage form. It can also be used in the quality control of bulk manufacturing of presented API’s.

## Supplementary Information

Below is the link to the electronic supplementary material.

Supplementary file1 (DOCX 228 KB)

## Data Availability

All data analyzed during this work are included in the submitted manuscript and supplementary file.
