# Supplementary material for: HPLC-Fluorescence Detection Method for Concurrent Estimation of Domperidone and Naproxen. Validation and Eco-Friendliness Appraisal Studies
Source: J Fluoresc. 2022 Dec 20;33(3):945–54. doi: 10.1007/s10895-022-03067-1 (PMC10140086; doi:10.1007/s10895-022-03067-1)
Supplement: Supplementary file 1 — Supplementary file1 (DOCX 228 KB) [file 10895_2022_3067_MOESM1_ESM.docx]

**HPLC-fluorescence detection method for concurrent estimation of domperidone and naproxen. Validation and eco-friendliness appraisal studies**

**Supplementary material**

**Figure S1 Calibration plot of the regression data of peak areas and the corresponding concentrations of DOM**

**Figure S2 Residuals plot of the regression data of peak area and the corresponding concentration of DOM**


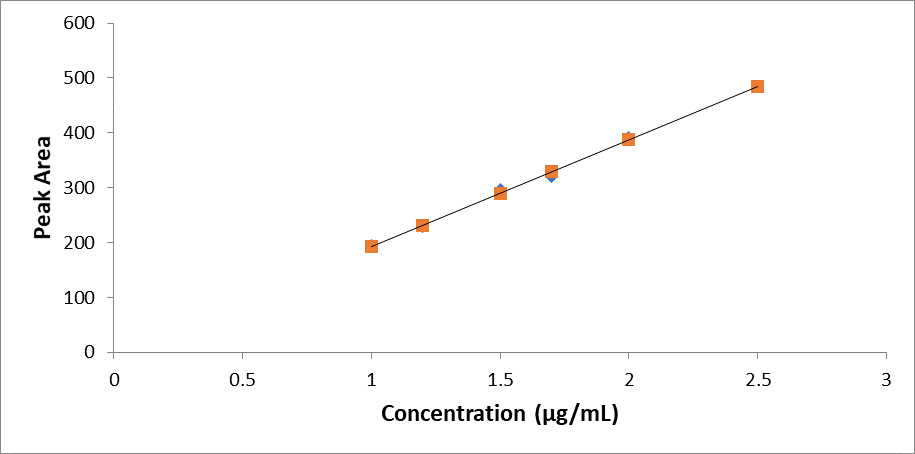


**Figure S3 Calibration plot of the regression data of peak areas and the corresponding concentrations of NAP**

**Figure S4 Residuals plot of the regression data of peak area and the corresponding concentration of NAP**


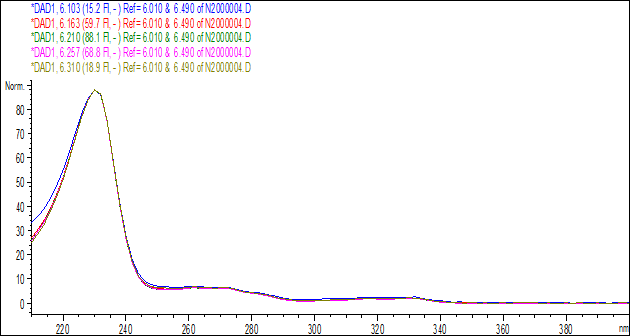


**NAP**


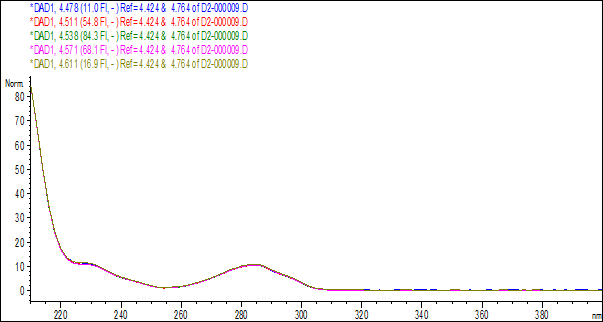


**DOM**

**Figure S5 Excitation purity spectra for DOM and NAP**


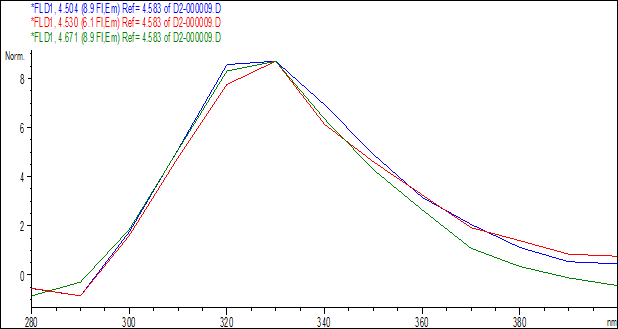


**DOM**


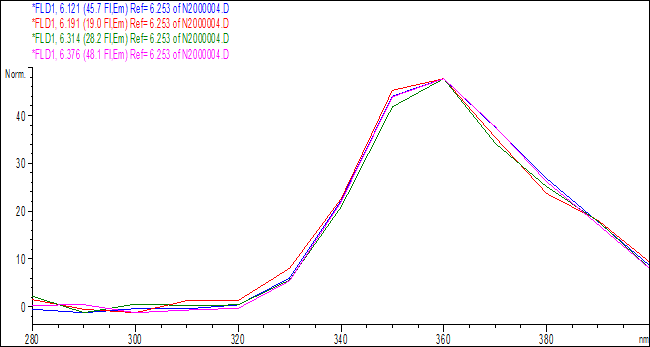


**NAP**

**Figure S6 Emission purity spectra for DOM and NAP**

**
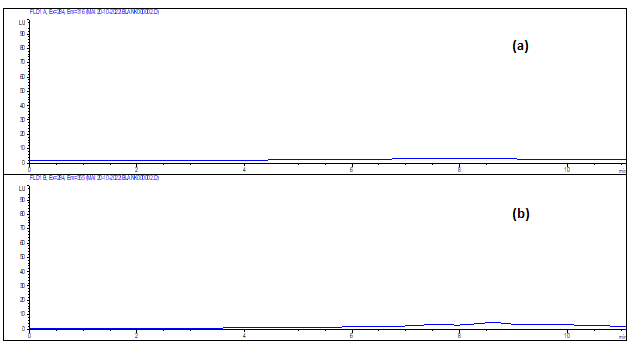
**

**Figure S7 HPLC Chromatogram of blank solution using the proposed method conditions at λex 284 nm, λem 316 nm (a) and at λem 355 nm (b), respectively**

**
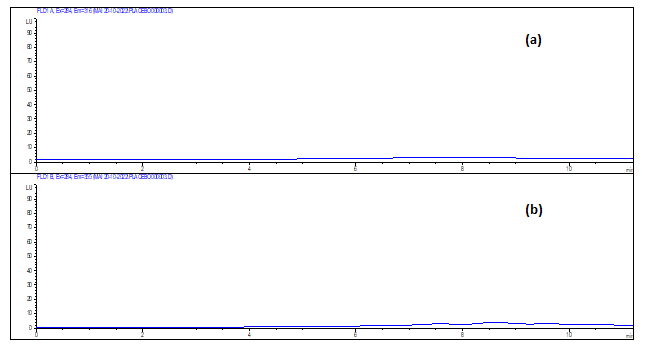
**

**Figure S8 HPLC Chromatogram of placebo solution using the proposed method conditions at λex 284 nm, λem 316 (a) nm and at λem 355 nm (b), respectively**

**Table S1 Evaluation of stability of working and sample solutions of the proposed HPLC-FD method**

| **Time (hour)** | **Working solutions** | | **Sample solutions** | |
| --- | --- | --- | --- | --- |
|  | **NAP**  **(2.5 µg/ mL)** | **DOM**  **(3.6 µg/ mL)** | **NAP**  **(2.0 µg/ mL)** | **DOM**  **(3.6 µg/ mL)** |
| **0** | 451.1 | 91.5 | 395.7 | 90.4 |
| **1** | 451.1 | 91.5 | 395.7 | 90.4 |
| **2** | 451.2 | 91.6 | 393.0 | 90.5 |
| **3** | 451.0 | 91.5 | 395.1 | 91.1 |
| **4** | 451.0 | 91.0 | 393.9 | 91.5 |
| **5** | 451.5 | 90.9 | 393.0 | 90.5 |
| **6** | 452.0 | 91.5 | 395.1 | 90.6 |
